# Decreased bone mass in adolescents with bone fragility fracture but not in young children: a case–control study

**DOI:** 10.3389/fendo.2023.1124896

**Published:** 2023-05-08

**Authors:** Velimir Matkovic, Prem Goel, Stacey L. Mobley, Nancy E. Badenhop-Stevens, Eun-Jeong Ha, Bin Li, Mario Skugor, Albert Clairmont

**Affiliations:** ^1^ Bone and Mineral Metabolism Laboratory, Departments of Physical Medicine and Rehabilitation (PMR), Medicine, and Nutrition, The Ohio State University, Columbus, OH, United States; ^2^ Department of Statistics, The Ohio State University, Columbus, OH, United States; ^3^ Food Sciences and Human Nutrition Department, University of Florida, Gainesville, FL, United States; ^4^ Department of Nutrition, Kent State University, Kent, OH, United States; ^5^ Department of Experimental Statistics, Louisiana State University, Baton Rouge, LA, United States; ^6^ Department of Endocrinology, Cleveland Clinic, Cleveland, OH, United States

**Keywords:** bone mass, fragility fracture, children, adolescents, vitamin D

## Abstract

**Background:**

The incidence of distal forearm fracture due to minimal/moderate trauma shows a bimodal distribution for age at event, with one peak occurring during early adolescence, in both boys and girls and the other one in postmenopausal females. The aim of this study was, therefore, to document whether the relationship between bone mineral density and fracture is different in young children compared with adolescents.

**Methods:**

A matched-pair, case–control study has been conducted to evaluate bone mineral density in 469 young children and 387 adolescents of both sexes, with/without fracture due to minimal/moderate trauma with assurance that the compared groups were equally susceptible to the outcome event. All fractures were radiographically confirmed. The study utilized bone mineral areal density of the total body, spine, hips, and forearm; volumetric bone mineral density of the forearm; and metacarpal radiogrammetry measurements. The study controlled for skeletal development, bone geometry, body composition, hand grip strength, calcium intake, and vitamin D status.

**Results:**

Adolescents with distal forearm fracture have reduced bone mineral density at multiple skeletal regions of interest. This was documented by the bone mineral areal density measurements at multiple skeletal sites (p < 0.001), volumetric bone mineral density measurements of the forearm (p < 0.0001), and metacarpal radiogrammetry (p < 0.001). Adolescent females with fracture had reduced cross-sectional areas of the radius and metacarpals. The bone status of young female and male children with fracture was no different to its controls. Increased body fatness was more prevalent among fracture cases than in controls. Around 72% of young female and male children with fracture had serum 25-hydroxyvitamin D levels below the threshold of 31 ng/ml, compared with only 42% of female controls and to 51% of male controls.

**Conclusions:**

Adolescents with bone fragility fracture had reduced bone mineral density at multiple skeletal regions of interest, whereas this was not the case with younger children. The results of the study may have implications for the prevention of bone fragility in this segment of the pediatric population.

## Introduction

The incidence of distal forearm fracture due to moderate trauma shows a bimodal distribution for age at event, with one peak occurring during adolescence, in both boys and girls, and the other one in postmenopausal women (1–5) as the consequence of type I osteoporosis (6). This trend is present even with the exclusion of a “green stick” fracture, typical for bone growth (5). Information about bone fragility fractures in children has received little attention in the public media as compared with adults. It is true that there is no mortality associated with a broken forearm and morbidity is minimal; however, the healthcare cost for its treatment is not small (7). In addition, the incidence of this type of fracture in children has been constantly increasing over the last few decades for reasons currently unknown (8, 9) and the total number of fractures is expected to rise due to the increasing number of children under age 18 years in the distant future (Population Projections for the United States 2017-2060, U.S. Census Bureau. Population Division, Washington, DC. www.census.gov).

It has been postulated that the interaction between endogenous (bone deficiency of growth due to a delay in endosteal apposition of bone) and exogenous (nutrition, physical activity) factors may play a significant role, compromising bone mass and strength leading to bone fragility (3, 10, 11). Several studies documented lower bone mineral areal and volumetric density measurements in children with fracture (12–27); however, no study separately evaluated bone status in younger children with fracture when the bone accretion is relatively slow as compared with adolescents who do have the highest rate of bone modeling and the highest number of bone fragility fractures. It was previously reported that the bone accrual rate was lower in girls who sustained bone fragility fracture while undergoing pubertal progression (28). To address these issues, we conducted a case–control study in young children and adolescents with distal forearm fracture due to minimal to moderate trauma, as well as in the normal healthy controls without fracture, matched to age, sex, ethnicity, and school belonging.

## Materials and methods

### Study design and study population

A matched-pair, case–control study with internal controls has been conducted with assurance that the compared groups were clinically similar and equally susceptible to trauma and bone fragility. The study, therefore, required the selection of the case and control groups from the same non-hospital community school roster (29). Young children and adolescents of both sexes and of all ethnic backgrounds who sustained a fracture of the distal forearm due to minimal/moderate trauma were recruited into the study. Young patients were recruited from pediatric and adult orthopedic services throughout central Ohio over a 4-year interval. All fractures were confirmed by radiographs. Each fracture case was matched with the appropriate control case without fracture. The search for the control subject proceeded immediately following the recruitment of a fracture case. Matching was done by chronological age at the time of the bone mass measurement, sex, ethnicity, and school belonging either public or private. If the appropriate control match was not found in the same classroom, the search for the matched subject proceeded within the same grade in his/her school (52% of matching accomplished this way), and finally in the same grade within his/her school district. Selecting the controls from the same classroom/school/school district was important because it provided powerful assurance that control cases had a similar socioeconomic status and were exposed as much as possible to the same living conditions, risk of trauma, and bone fragility, as the fracture cases. This approach was considered superior to the recruitment strategies based on the convenient patient samples selected from hospital populations. Such a recruitment of the control cases would be prone to error due to a higher risk of hospital admissions for various reasons, which could ultimately affect the measured outcomes (29).

Skeletal age has been shown to be the best clinical determinant of bone mineral density during growth (30). However, it was not selected as the matching criteria for the recruitment of the control cases as this would require numerous hand X-rays in healthy children, which could compromise the study execution due to problems associated with irradiation, delays in IRB approval and recruitment, and extra costs. As this study also included young children, the pubertal staging as the matching criteria for the recruitment of the control cases was not selected either. Both clinical determinants of bone mineralization, however, were controlled for by the study design.

After minors and their parents gave written informed consent, each participant was referred to the Ohio State University, Bone and Mineral Metabolism Laboratory, for further evaluation. Each participant in the study had a detailed medical history; trauma history; dietary history; physical examination including blood pressure measurement and pubertal status determination; blood draw; basic anthropometry; handgrip strength evaluation; hand X-ray for skeletal age and radiogrammetry of the metacarpal bones; body composition; bone mass measurements of the total body, forearm, spine, and hip by dual-energy X-ray absorptiometry (DXA); and forearm measurements at the proximal and distal radii by pQCT (peripheral quantitative computerized tomography).

We estimated the required sample size of around 450/group to detect 2.5% change in mean of the distal forearm bone mineral density (BMD with power fixed at 0.95 and the ratio of mean and sigma at 8). The total number of participants in the study was 856: 458 fracture cases, 248 of which are young children (104 females, 144 males) and 210 in adolescents (82 females, 128 males), and 398 controls, 221 of which are young children (93 females, 128 males) and 177 adolescents (72 females, 105 males). The discrepancy between the fracture cases and controls (n = 60) was in part due to a difficulty in matching for ethnicity and school belonging. However, the comparisons of the main outcome variables between the fracture cases having the appropriate controls (N = 398) and fracture cases without controls (N = 60) did not reveal any statistically significant difference between the two groups based on the Kolmogorov–Smirnov goodness-of-fit test. The study protocol was approved by the Biomedical Sciences Institutional Review Boards at The Ohio State University, Nationwide Children’s Hospital, and local Medical Centers in central Ohio.

### Patients

The operational diagnostic criteria for definition and detection of the disease event under study included all distal forearm (radius and ulna) fractures confirmed by an X-ray, caused by minimal to moderate trauma, and with body weight of participants >25 kg. The following fractures were included: distal radius and ulna [physeal, metaphyseal (torus, greenstick, complete), Galeazzi fracture] and distal shaft of the radius and ulna (below 1/3 of the forearm length). The degree of trauma was classified as moderate or severe based on the following criteria: 1) moderate trauma: injuries caused by falls exerted by the same individual—fall to the ground from a standing level and on the same level; most of the sports-related injuries including ball sports, skating, wrestling, and gymnastics but not falls from heights. This included skateboard and roller blading injuries, falling from less than a meter of height (stools, chairs, beds, bicycle, slides, and similar playing equipment and a child being hit by a bicycle); 2) severe trauma: falling from a height of more than a meter including most falls from windows, roofs, and others, and all traffic/motorized vehicle accidents. The severe trauma victims and children with history of multiple fractures were not eligible to participate in the study. All fracture cases had to be ambulatory (before and after trauma), free of any metabolic bone disease or other chronic disease, and not taking corticosteroids, thyroid, and other medications known to affect bone mass. Individuals with fracture who required internal or external fixation with metal rods were not eligible to participate; instrumentation could influence whole-body bone mineral density measurement by DXA, and in most such cases, severe trauma was involved. Body weight >25 kg was selected as the inclusion criteria to allow for using only adult software for body composition and bone mass measurement. This eliminated very young children (less than 6 years of age) from participation in the study; however, according to our preliminary data and data of others, the frequency of forearm fractures in this age group is relatively low (1, 3, 5).

Most of the fractures (62%) happened during the time interval from May to October, and less so (38%) from November to April of each year of recruitment. The ethnic distribution of the fracture cases (88% Caucasian, 8% African American, 0.7% Hispanic, 0.2% Asian, 1.5% African American-Caucasian, 1.6% other mixed racial) corresponded to the ethnic diversity of the central Ohio population. The median time since fracture was 69.5 days, with 3/4 of participants evaluated within 154 days since trauma. The time since fracture was not considered critical for bone mass measurements as this type of fracture healed quickly and did not lead to a significant restriction in the activities of daily living and the fractured arm was not measured.

### Controls

Control subjects come from the same population at risk of the disease or condition being studied (internal control), in this case the risk of trauma and bone fragility leading to fracture. Controls, therefore, were exposed to a similar risk/opportunity for fracture. Excluding fracture, other eligibility criteria selected for cases applied to controls as well.

### Clinical data and anthropometry

Physical examination including blood pressure measurements and detailed medical (menstrual history in females, medication use) and trauma history were taken by trained personnel. Blood pressure was measured using a Hawksley random-zero sphygmomanometer with three consecutive measurements. Body weights were recorded in kilograms to the nearest 10th in normal indoor clothing without shoes. Standing height was recorded without shoes on a portable stadiometer. Each subject stood with his/her back against the axis of the measuring rod with the mandible plan parallel to the floor. Values were recorded to the nearest 10th of a centimeter. Pubertal stage based on the development of secondary sexual characteristics was evaluated by self-assessment with marking corresponding illustrations of sexual development (31, 32). This was helped by parents for younger children. Handgrip strength was evaluated by a Jamar hydraulic dynamometer (Sammons Preston, Bolingbrook, IL), and the average of three recordings was used. Skeletal age was determined based on standardized hand X-rays *via the* FELS method (30, 33).

Blood draw for vitamin D status determination was obtained in a non-fasting state, ensuring that the blood draws of the fracture case and its control were done in the same season. Serum samples were stored in a freezer at –80°C, and 25-hydroxy vitamin D [25(OH)D] was simultaneously analyzed at the end of the study by a radioimmunoassay (RIA) kit (DiaSorin, Stillwater MN; inter-assay CV 11%; intra-assay CV 10%; reproducibility of 95% by running 10 samples in duplicate). Dietary intake including supplementation was assessed by a 3-day dietary record and analyzed using Nutritionist III software (version 8.5 for Macintosh, Hearst Corp) as done before (34). The results were presented as a mean daily intake of energy, protein, carbohydrates, fat, calcium, phosphorus, zinc, vitamin D, and soda beverages. Calcium intakes from various sources (dairy and combination foods) were analyzed separately.

### Bone mineral density measurements and bone morphometry

Each participant had a bone mineral areal density measurement (BMD g/cm^2^) of the whole body, hip, spine, and forearm by the DXA technique (GE Lunar Prodigy, v5.6, Madison, WI). The DXA measurements were taken in a standard position using medium-speed scan. Data about body composition were obtained from the whole-body scan including body fat, lean body mass, and total body bone mineral content. Non-fractured forearm was measured at the proximal (1/3) and distal (10 mm) measuring sites. Volumetric bone mineral density measurements of the non-fractured radius at the proximal sites (33%) and distal sites (10%) were performed using a pQCT (Norland Stratec XCT-2000) densitometer with contour, peel, and separation modes of 2, 2, 2, respectively, as described before (35). The comparable arms were measured by both techniques in the control population. The precision errors for bone mass measurements in our Laboratory were previously reported, and they were as follows: 0.7% for the whole body, 1.0% for the spine, 2% for the hip, and 0.8% for the forearm sites (32, 34).

The hand X-rays of the non-fractured arm and the corresponding side in the controls served for radiogrammetry of the metacarpals to evaluate changes at the periosteal and endosteal bone envelopes. Radiogrammetry was performed by the automated X-posure System (Pronosco A/S, Voedboek, Denmark) (34). Measurements were made of the external (T) and internal (M) metacarpal diameters at multiple points of the second, third, and fourth metacarpal bones. From these primary measurements, the average cross-sectional area parameters were calculated including total area (TA), medullary area (MA), and cortical area (CA=TA-MA). The area estimates are based upon the assumptions of a circular geometry. The CVs for the static and repositioned measurements were 0.0% and 0.2% for bone width and 0.5% and 0.6% for cortical thickness, respectively, as reported (34).

### Statistical analysis

Hotelling’s T^2^ statistics was used to account for the multiplicity in comparing two groups with multiple variables and to simultaneously test the equality of the means of a paired multivariate data set of fracture cases and its age-matched controls (36, 37). This was done separately for female and male subjects of different age: young female children ≤11 years (age range 6–11 years), young male children ≤12 years (age range 6.7–12 years); female adolescents >11 years (age range 11.1–17.2 years), male adolescents >12 years (age range 12.1–17.6). The age ≤11 years was selected as a cutoff point between young female children and adolescent females based on the results of a 7-year longitudinal study conducted in a cohort of 354 young females recruited from the same school districts in central Ohio (34). The maximal bone mineral accretion rate in the cohort with the highest levels of serum and urine bone biomarkers was present at the age 12.8 ± 0.9 years (34, 38). A 1-year difference in the age classification between females and males has been based on a delay in sexual maturation in boys.

The simultaneous tests for mean vectors was done for the following biologically relevant subsets of variables in each age/sex/subgroup including the following: demography, skeletal age, pubertal stage, hand grip strength, and blood pressure; anthropometry, body composition, and bone mineral areal density variables of various skeletal regions of interest and volumetric density of the proximal and distal radii with cross-sectional bone geometry; radiogrammetry of the metacarpal bones; and dietary intake of major nutrients. Empirical cumulative distribution function (ecdf) and a two-sample Kolmogorov–Smirnov test to quantify the distance between the two samples were used to present and analyze relative body fat content (pBF) and 25(OH)D blood levels within the groups (p value and test statistic reported). Most of the exploratory analysis of data was performed *via* standard statistical and graphical methods using commercially available statistical packages such as Data Desk, SAS, and Open Source Software Package R, MVTests, for Hotelling’s T-square analysis.

## Results

### Demographic and clinical data

The chronological age of the fracture cases and its controls was practically the same within the groups due to study design. The coefficient of correlation between the chronological age of the fracture cases and its controls was 0.999. The coefficients of correlation between the chronological age and skeletal age of the fracture cases and the controls were 0.905 and 0.903, respectively. In addition, the coefficient of correlation between the skeletal age of the fracture cases and its controls was also very high at 0.823, indicating a very close similarity in skeletal development between the groups. Young female children with fracture were on average 9.3 ± 1.3 years old (mean ± SD), and young male children with fracture were 9.8 ± 1.3 years old. The corresponding control groups had an average age of 9.3 ± 1.2 and 9.9 ± 1.4 years, respectively. Adolescent women with fracture were on average 12.6 ± 1.4 years old, and adolescent men had an average age of 13.8 ± 1.2 years. The corresponding adolescent controls had an average age of 12.6 ± 1.4 and 13.8 ± 1.2 years, respectively.

Even though the control cases were matched based on chronological age, it should be noted that paired Hotelling’s T^2^ statistics did not reveal a statistically significant difference in skeletal age, pubertal developmental status, handgrip strength, and blood pressure between the fracture cases and its controls, either among young children or adolescents (**Table 1a**). This provided powerful assurance of a similarity in clinical features of the selected groups. Stature, body weight, BMI, and body composition variables (body fat, percent body fat, lean body mass, and total body bone mineral content) were no different between fracture cases and its controls in young children of both sexes (**Table 1b**). Among female adolescents, no single individual comparison of these variables between the groups was statistically significant, although the overall test for difference in the anthropometry variables of the fracture cases *versus* controls showed a significant difference, presumably due to correlation between the variables. This seeming contradiction can occur sometimes simply because the overall test examines the equality of all possible linear functions of these variables, and one of these may be significantly different. The anthropometry of adolescent men was no different between the groups (**Table 1b**).

**Table 1:** Hotelling’s T^2^ statistics for skeletal age, pubertal development, handgrip, and blood pressure (a) and anthropometry variables (b) of young children and adolescents with bone fragility fracture and its age-matched controls.

(a)			
Young female children (N = 88 pairs) T^2^ statistics:3.306; p value: 0.792	Fracture cases Mean ± SD	Controls Mean ± SD	95% C.I.
Skeletal age (years)	10.3 ± 1.8	10.2 ± 1.6	-0.5, 0.7
Pubertal stage breast	1.4 ± 0.6	1.5 ± 0.7	-0.4, 0.2
Pubertal stage pubes	1.3 ± 0.6	1.3 ± 0.7	-0.4, 0.3
Handgrip (kg)	14.2 ± 4.1	13.7 ± 3.2	-1.4, 2.4
Blood pressure systolic (mmHg)	102.2 ± 7.4	101.1 ± 8.5	-3.3, 5.5
Blood pressure diastolic (mmHg)	67.5 ± 4.9	66.4 ± 7.2	-2.4 ± 4.5
Young male children (N = 119 pairs) T^2^ statistics: 10.389; p value: 0.083			
Skeletal age (years)	10.0 ± 2.0	10.1 ± 1.9	-0.6, 0.4
Pubertal stage pubes	1.4 ± 0.7	1.3 ± 0.6	-0.1, 0.3
Handgrip (kg)	16.2 ± 4.3	16.8 ± 4.5	-2.2, 0.9
Blood pressure systolic (mmHg)	101.5 ± 7.5	101.7 ± 8.9	-3.9, 3.5
Blood pressure diastolic (mmHg)	66.9 ± 5.8	68.1 ± 8.1	-4.4 ± 2.0
Female adolescents (N = 68 pairs) T^2^ statistics:13.666; p value: 0.065			
Skeletal age (years)	13.1 ± 1.4	13.3 ± 1.7	-0.8, 0.6
Pubertal stage breast	2.6 ± 1.0	2.7 ± 1.1	-0.6, 0.5
Pubertal stage pubes	3.2 ± 1.5	3.1 ± 1.5	-0.7, 0.9
Handgrip (kg)	20.5 ± 5.8	21.9 ± 5.8	-4.3, 1.5
Blood pressure systolic (mmHg)	107.7 ± 7.7	105.2 ± 7.8	-2.2, 7.0
Blood pressure diastolic (mmHg)	69.4 ± 6.6	68.9 ± 7.3	-4.0 ± 5.0
Male adolescents (N = 103 pairs) T^2^ statistics: 2.404; p value: 0.804			
Skeletal age (years)	14.6 ± 1.3	14.6 ± 1.8	-0.5, 0.7
Pubertal stage pubes	3.5 ± 0.9	3.4 ± 1.1	-0.2, 0.5
Handgrip (kg)	29.9 ± 8.5	29.2 ± 8.4	-2.7, 4.0
Blood pressure systolic (mmHg)	109.0 ± 7.7	108.1 ± 9.2	-3.2, 5.1
Blood pressure diastolic (mmHg)	70.5 ± 4.8	69.8 ± 6.7	-2.2 ± 3.5

(b)			
Young female children (N = 92 pairs) T^2^ statistics:10.19; p value: 0.233	Fracture cases Mean ± SD	Controls Mean ± SD	95% C.I.
Height (cm)	138.1 ± 8.4	137.7 ± 8.3	-3.2, 4.1
Weight (kg)	37.5 ± 9.9	34.8 ± 9.0	-2.7, 7.9
BMI (kg/m^2^)	19.5 ± 3.9	18.2 ± 3.4	-0.8, 3.3
Body fat (g)	9,983 ± 7,082	8,246 ± 6,072	-2,098, 5,571
Body fat %	26.0 ± 10.6	23.5 ± 10.1	-4.0, 8.9
Lean body mass (g)	24,884 ± 3,993	23,941 ± 3,552	-894, 2,780
Total body BMC (g)	1,204 ± 273	1,150 ± 242	-81, 189
Young male children (N = 124 pairs) T^2^ statistics: 10.708; p value: 0.19			
Height (cm)	141.0 ± 9.7	141.0 ± 8.8	-2.7, 2.7
Weight (kg)	38.3 ± 12.2	37.5 ± 10.9	-4.0, 5.7
BMI (kg/m^2^)	19.0 ± 3.9	18.7 ± 3.8	-1.6, 2.2
Body fat (g)	8,701 ± 7,948	7,381 ± 6,602	-2,011, 4,651
Body fat %	21.4 ± 11.5	19.0 ± 10.7	-2.9, 7.7
Lean body mass (g)	26,761 ± 4,881	27,072 ± 4,601	-2,056, 1,433
Total body BMC (g)	1,283 ± 351	1,289 ± 318	-125, 114
Female adolescents (N=71 pairs) T^2^ statistics:32.598; p value: 0.001			
Height (cm)	155.4 ± 9.3	155.7 ± 8.8	-5.2, 4.5
Weight (kg)	50.4 ± 15.3	49.6 ± 15.0	-8.5, 10.0
BMI (kg/m^2^)	20.6 ± 4.6	20.2 ± 5.0	-2.7, 3.4
Body fat (g)	13,886 ± 9,532	12,432 ± 9,619	-4,853, 7,760
Body fat %	27.2 ± 10.1	24.4 ± 10.0	-3.7, 9.3
Lean body mass (g)	33,382 ± 6,722	33,899 ± 6,487	-4,027, 2,994
Total body BMC (g)	1,739 ± 555	1,871 ± 509	-393, 129
Male adolescents (N=108 pairs) T^2^ statistics:13.429; p value: 0.093			
Height (cm)	165.6 ± 10.7	163.9 ± 10.4	-2.8, 6.2
Weight (kg)	62.8 ± 17.3	57.0 ± 14.6	-2.2, 13.9
BMI (kg/m^2^)	22.6 ± 4.9	20.9 ± 3.7	-0.6, 4.1
Body fat (g)	14,913 ± 11,758	10,703 ± 7,604	-1,270, 9,690
Body fat %	23.7 ± 12.8	18.6 ± 9.7	-2.1, 10.4
Lean body mass (g)	44,489 ± 9,250	42,749 ± 9,605	-2,262, 5,741
Total body BMC (g)	2,279 ± 570	2,148 ± 562	-130, 392

**Figure 1:**
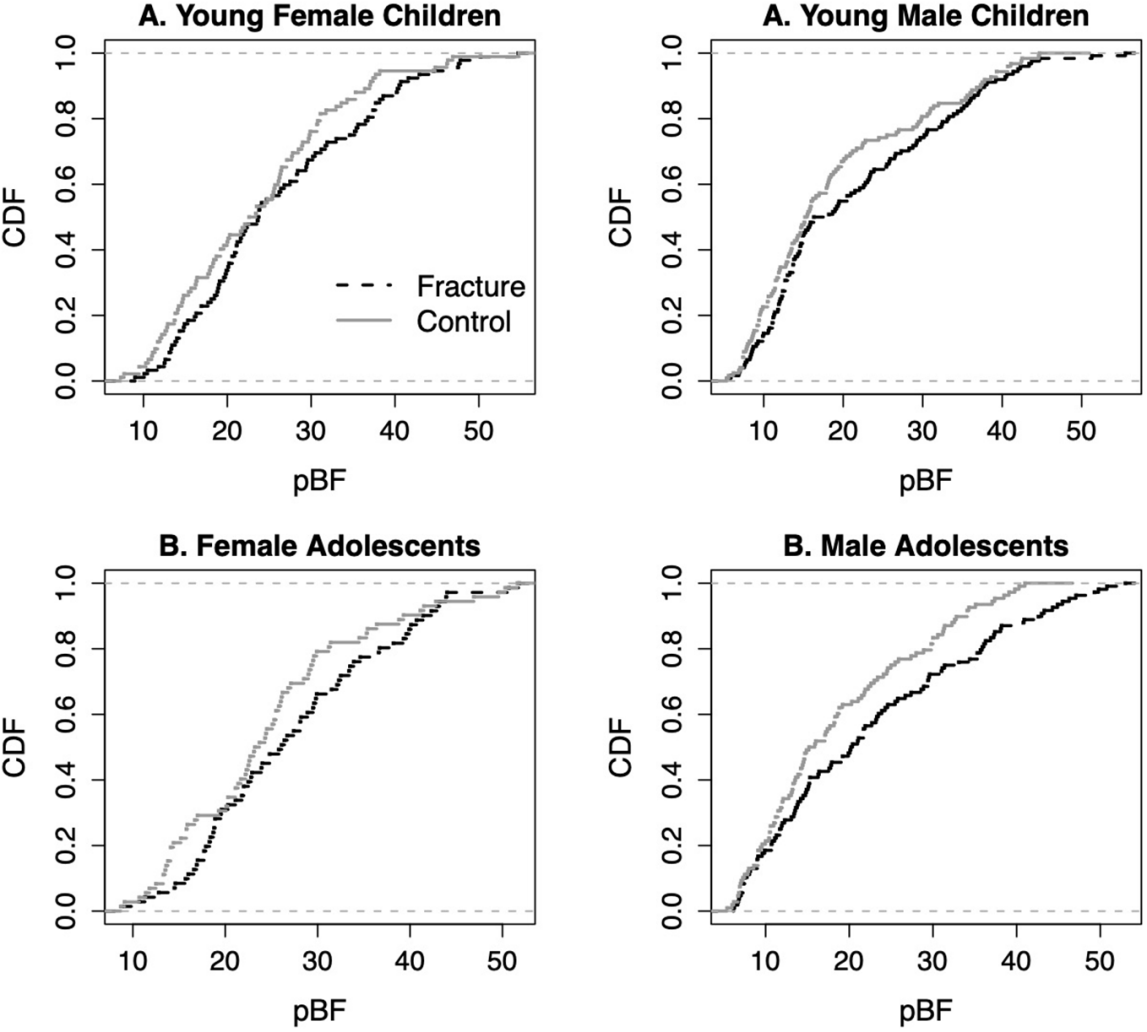
Empirical cumulative distribution functions (cdf) of percent body fat (pBF) in young children and adolescents with fracture and its controls.

Even though the mean body fat content and percent body fat of the fracture cases as compared with the control groups were not different (**Table 1b**), a close examination by the empirical cumulative distribution function (ecdf) of the percent body fat (pBF) in young children and adolescents with fracture and its controls showed that there were always higher proportions of the fracture cases above a certain point of the percent body fat as compared with the controls (**Figure 1**).

### Bone mineral density measurements and bone morphometry

Bone mineral areal density variables at multiple skeletal regions of interest, volumetric bone mineral density of the proximal and distal radius with cross-sectional bone geometry, and morphometry of the metacarpal bones were not statistically different in young children with fracture as compared with their counterparts without fracture (**Tables 2a, b**, **3**). However, all the bone mineral areal density parameters at multiple skeletal sites and volumetric bone mineral density variables of the proximal and distal radii including trabecular bone mass were significantly reduced in female adolescents with fracture as compared with the controls (**Tables 2a, b**). In addition, adolescent women with fracture had significantly lower cortical bone mass at the proximal radius and the metacarpals with slightly reduced cross-sectional areas at both skeletal regions of interest compared with their controls (**Table 3**). Comparing the bone mineral areal density variables among adolescent men with and without the fracture revealed a statistically significant difference between the groups, although there was no significant difference in any single parameter; forearm and hip bone mineral areal density measurements were lower in the men with fracture (**Table 2a**). Adolescent men with fracture were also significantly different from their controls with regard to the volumetric bone mineral density parameters, at both the proximal and distal measuring sites of the radius. All measurements were lower in the fracture cases indicating bone deficiency within a bone as an organ (**Table 2b**). The cross-sectional areas of radius at two locations were higher in adolescent men with fracture as compared with the controls, whereas morphometry of the metacarpals was not different between the groups (**Table 3**).

**Table 2:** Hotelling’s T^2^ statistics for bone mineral areal (a) and volumetric (b) density of various skeletal regions of interest of young children and adolescents with bone fragility fracture and its age-matched controls.

(a)			
Young female children (N = 91 pairs) T^2^ statistics: 3.223; p value: 0.801	Fracture cases Mean ± SD	Controls Mean ± SD	95% C.I.
Distal radius BMD (g/cm^2^)	0.278 ± 0.052	0.273 ± 0.040	-0.018, 0.030
Proximal radius BMD (g/cm^2^)	0.480 ± 0.048	0.469 ± 0.047	-0.013, 0.034
Femur neck BMD (g/cm^2^)	0.813 ± 0.524	0.830 ± 0.648	-0.347, 0.313
Femur trochanter BMD (g/cm^2^)	0.712 ± 0.512	0.731 ± 0.646	-0.347, 0.309
Spine L_2-4_ BMD (g/cm^2^)	0.757 ± 0.090	0.746 ± 0.093	-0.039, 0.062
Total body BMD (g/cm^2^)	0.876 ± 0.062	0.870 ± 0.058	-0.024, 0.037
Young male children (N = 121 pairs) T^2^statistics: 6.208; p value: 0.434			
Distal radius BMD (g/cm^2^)	0.287 ± 0.037	0.294 ± 0.036	-0.023, 0.010
Proximal radius BMD (g/cm^2^)	0.479 ± 0.059	0.491 ± 0.050	-0.035, 0.011
Femur neck BMD (g/cm^2^)	0.831 ± 0.153	0.891 ± 0.455	-0.225, 0.104
Femur trochanter BMD (g/cm^2^)	0.731 ± 0.149	0.785 ± 0.444	-0.212, 0.104
Spine L_2-4_ BMD (g/cm^2^)	0.727 ± 0.080	0.734 ± 0.086	-0.040, 0.027
Total body BMD (g/cm^2^)	0.886 ± 0.070	0.893 ± 0.071	-0.033, 0.018
Female adolescents (N = 71 pairs) T^2^ statistics: 27.2; p value: 0.001			
Distal radius BMD (g/cm^2^)	0.292 ± 0.063	0.329 ± 0.064	-0.068, -0.006
Proximal radius BMD (g/cm^2^)	0.541 ± 0.068	0.578 ± 0.073	-0.071, -0.004
Femur neck BMD (g/cm^2^)	0.868 ± 0.144	0.950 ± 0.148	-0.161, -0.003
Femur trochanter BMD (g/cm^2^)	0.743 ± 0.135	0.823 ± 0.143	-0.157, -0.003
Spine L_2-4_ BMD (g/cm^2^)	0.896 ± 0.168	0.994 ± 0.186	-0.176, -0.021
Total body BMD (g/cm^2^)	0.958 ± 0.117	1.019 ± 0.117	-0.114, -0.008
Male adolescents (N = 107 pairs) T^2^ statistics: 17.483; p value: 0.015			
Distal radius BMD (g/cm^2^)	0.334 ± 0.052	0.348 ± 0.063	-0.040, 0.012
Proximal radius BMD (g/cm^2^)	0.587 ± 0.070	0.599 ± 0.075	-0.043, 0.020
Femur neck BMD (g/cm^2^)	0.973 ± 0.116	0.998 ± 0.139	-0.086, 0.035
Femur trochanter BMD (g/cm^2^)	0.865 ± 0.114	0.879 ± 0.148	-0.075, 0.046
Spine L_2-4_ BMD (g/cm^2^)	0.942 ± 0.149	0.944 ± 0.166	-0.067, 0.063
Total body BMD (g/cm^2^)	1.034 ± 0.100	1.032 ± 0.107	-0.043, 0.047

(b)			
Young female children (N = 63 pairs) T^2^ statistics: 2.655; p value: 0.642	Fracture cases Mean ± SD	Controls Mean ± SD	95% C.I.
Proximal radius total density (mg/cm^3^)	819.9 ± 65.5	823.9 ± 63.3	-43.2, 35.1
Proximal radius cortical density (mg/cm^3^)	1,083.0 ± 37.2	1,086.4 ± 31.2	-22.1, 15.3
Distal radius total density (mg/cm^3^)	282.0 ± 37.0	283.2 ± 34.3	-19.8, 17.3
Distal radius trabecular density (mg/cm^3^)	206.0 ± 32.5	211.3 ± 30.3	-23.1, 12.5
Young male children (N=91 pairs) T^2^ statistics: 8.318; p value: 0.1			
Proximal radius total density (mg/cm^3^)	804.5 ± 76.3	815.8 ± 57.2	-44.1, 21.4
Proximal radius cortical density (mg/cm^3^)	1,078.2 ± 43.0	1,087.3 ± 32.6	-26.1, 7.8
Distal radius total density (mg/cm^3^)	288.6 ± 37.6	298.1 ± 30.6	-25.5, 6.5
Distal radius trabecular density (mg/cm^3^)	198.6 ± 33.1	209.0 ± 28.3	-24.0, 3.2
Female adolescents (N=53 pairs) T^2^ statistics: 30.554; p value: 0			
Proximal radius total density (mg/cm^3^)	854.8 ± 63.5	892.5 ± 67.5	-76.3, 0.8
Proximal radius cortical density (mg/cm^3^)	1,105.2 ± 45.8	1,126.3 ± 37.1	-44.0, 1.8
Distal radius total density (mg/cm^3^)	269.0 ± 38.8	303.6 ± 42.2	-59.3, -9.9
Distal radius trabecular density (mg/cm^3^)	183.9 ± 38.4	214.1 ± 31.5	-51.1, -9.4
Male adolescents (N=85 pairs) T^2^ statistics: 16.583; p value: 0.005			
Proximal radius total density (mg/cm^3^)	830.5 ± 59.2	855.8 ± 62.4	-51.9, 1.3
Proximal radius cortical density (mg/cm^3^)	1,088.5 ± 37.9	1,097.4 ± 32.4	-24.3, 6.5
Distal radius total density (mg/cm^3^)	288.6 ± 32.2	303.0 ± 37.0	-29.2, 0.4
Distal radius trabecular density (mg/cm^3^)	210.2 ± 30.2	220.5 ± 35.5	-26.2, 5.6

**Table 3:** Hotelling’s T^2^ statistics of cross-sectional bone geometry of the proximal and distal radius and metacarpal radiogrammetry of young children and adolescents with bone fragility fracture and its matched controls.

Young female children (N=63 pairs) T^2^ statistics: 5.34; p value: 0.172	Fracture cases Mean ± SD	Controls Mean ± SD	95% C.I.
Proximal radius TA (mm^2^)	74.6 ± 12.2	71.8 ± 10.3	-2.7, 8.3
Proximal radius CA (mm^2^)	49.1 ± 7.4	47.8 ± 6.8	-2.2, 4.7
Distal radius TA (mm^2^)	226.3 ± 43.8	228.2 ± 32.5	-20.4, 16.6
Young male children (N=91 pairs) T^2^ statistics: 4.258; p value: 0.252			
Proximal radius TA (mm^2^)	80.0 ± 14.0	79.6 ± 12.3	-4.9, 5.7
Proximal radius CA (mm^2^)	51.3 ± 8.7	52.0 ± 8.1	-3.8, 2.2
Distal radius TA (mm^2^)	239.4.0 ± 44.4	246.9 ± 46.3	-24.0, 9.1
Female adolescents (N=53 pairs) T^2^ statistics: 21.162; p value: 0.001			
Proximal radius TA (mm^2^)	87.0 ± 17.7	92.0 ± 16.8	-12.4, 2.4
Proximal radius CA (mm^2^)	59.9 ± 12.3	66.3 ± 12.0	-11.4, -1.3
Distal radius TA (mm^2^)	283.7 ± 54.2	291.3 ± 46.8	-32.7, 17.6
Male adolescents (N=85 pairs) T^2^ statistics: 9.284; p value: 0.034			
Proximal radius TA (mm^2^)	110.7 ± 19.8	105.7 ± 18.9	-2.8, 12.6
Proximal radius CA (mm^2^)	76.1 ± 14.1	75.1 ± 14.7	-4.6, 6.6
Distal radius TA (mm^2^)	364.9 ± 67.4	349.7 ± 57.9	-11.7, 42.1
Young female children (N=86 pairs) T^2^ statistics: 1.769; p value: 0.633			
Metacarpal total area (cm^2^)	0.384 ± 0.049	0.379 ± 0.050	-0.018, 0.029
Metacarpal cortical area (cm^2^)	0.228 ± 0.029	0.230 ± 0.033	-0.015, 0.012
Metacarpal cortical/total area	0.596 ± 0.062	0.610 ± 0.071	-0.043, 0.016
Young male children (N=114 pairs) T^2^ statistics: 4.849; p value: 0.196			
Metacarpal total area (cm^2^)	0.429 ± 0.064	0.441 ± 0.066	-0.037, 0.012
Metacarpal cortical area (cm^2^)	0.237 ± 0.036	0.246 ± 0.037	-0.021, 0.003
Metacarpal cortical/total area	0.556 ± 0.052	0.562 ± 0.052	-0.025, 0.014
Female adolescents (N=69 pairs) T^2^ statistics: 23.15; p value: 0.000			
Metacarpal total area (cm^2^)	0.407 ± 0.058	0.439 ± 0.050	-0.059, -0.006
Metacarpal cortical area (cm^2^)	0.267 ± 0.046	0.298 ± 0.045	-0.050, -0.012
Metacarpal cortical/total area	0.656 ± 0.075	0.678 ± 0.072	-0.051, 0.007
Male adolescents (N=103 pairs) T^2^ statistics: 1.614; p value: 0.664			
Metacarpal total area (cm^2^)	0.536 ± 0.074	0.527 ± 0.076	-0.020, 0.038
Metacarpal cortical area (cm^2^)	0.333 ± 0.055	0.333 ± 0.058	-0.019, 0.020
Metacarpal cortical/total area	0.623 ± 0.069	0.633 ± 0.079	-0.035, 0.015

### Nutrition and vitamin D status

The dietary intake of various nutrients was not statistically different among the fracture cases and their controls, among either young children or adolescents of both sexes (**Table 4**). The determination of the vitamin D status of the study participants showed a seasonal variation in the serum 25(OH)D concentration at the time of blood draw. The serum levels of 25(OH)D among the study participants recruited during the time interval from May to October were as follows: (mean ± SD) 30.7 ± 11.4 ng/ml in the fracture group and 33.8 ± 9.6 ng/ml among the controls. Participants who were recruited during the interval from November to April had statistically significant lower serum 25(OH)D concentrations (24.1 ± 7.3 ng/ml fracture group, 25.7 ± 7.5 ng/ml control group; p < 0.0001) comparable with the subjects recruited during the summer season.

**Table 4:** Hotelling’s T^2^ statistics for energy and nutrients intake of young children and adolescents with bone fragility fracture and its age-matched controls.

Young female children (N=48 pairs) T^2^ statistics: 12.291; p value: 0.567	Fracture cases Mean ± SD	Controls Mean ± SD	95% C.I.
Energy (kcal/day)	1,894 ± 419	1,888 ± 503	-495, 507
Protein (g/day)	68 ± 17	68 ± 18	-19,19
Carbohydrates (g/day)	256 ± 60	261 ± 73	-79, 69
Fat (g/day)	70 ± 21	66 ± 22	-20, 28
Total calcium (mg/day)	1,113 ± 391	1,123 ± 453	-471, 452
Calcium dairy (mg/day)	604 ± 341	639 ± 417	-488, 418
Calcium combination foods (mg/day)	151 ± 147	115 ± 101	-109, 180
Phosphorus (mg/day)	1,233 ± 363	1,236 ± 406	-452, 444
Zinc (mg/day)	11 ± 5	11 ± 5	-6, 5
Vitamin D (mcg/day)	7 ± 4	8 ± 5	-5, 4
Soda beverage (can/day)	0.4 ± 0.6	0.4 ± 0.4	-0.7, 0.6
Young male children (N=84 pairs) T^2^ statistics: 17.418; p value: 0.195			
Energy (kcal/day)	2,244 ± 529	2,088 ± 448	-223, 534
Protein (g/day)	85 ± 26	76 ± 22	-10, 28
Carbohydrates (g/day)	296 ± 71	279 ± 63	-36, 69
Fat (g/day)	84 ± 26	78 ± 22	-11, 23
Total calcium (mg/day)	1,283 ± 436	1,238 ± 485	-329, 419
Calcium dairy (mg/day)	722 ± 377	714 ± 449	-321, 338
Calcium combination foods (mg/day)	173 ± 142	139 ± 118	-58, 126
Phosphorus (mg/day)	1,478 ± 447	1,407 ± 458	-292, 433
Zinc (mg/day)	13 ± 6	12 ± 6	-4, 5
Vitamin D (mcg/day)	9 ± 6	9 ± 6	-6, 4
Soda beverage (can/day)	0.7 ± 0.8	0.6 ± 0.6	-0.4, 0.6
Female adolescents (N=42 pairs) T^2^ statistics:11.091; p value: 0.673			
Energy (kcal/day)	2,110 ± 633	2,072 ± 515	-605, 682
Protein (g/day)	73 ± 25	72 ± 17	-22, 23
Carbohydrates (g/day)	281 ± 80	281 ± 73	-94, 92
Fat (g/day)	81 ± 33	76 ± 25	-26, 36
Total calcium (mg/day)	1,153 ± 519	1,082 ± 433	-411, 552
Calcium dairy (mg/day)	597 ± 357	600 ± 328	-373, 369
Calcium combination foods (mg/day)	163 ± 155	153 ± 111	-107, 126
Phosphorus (mg/day)	1,314 ± 514	1,306 ± 390	-460, 478
Zinc (mg/day)	11 ± 6	12 ± 6	-8, 8
Vitamin D (mcg/day)	7 ± 5	7 ± 5	-6, 6
Soda beverage (can/day)	0.8 ± 0.9	0.7 ± 0.6	-0.7, 0.9
Male adolescents (N=63 pairs) T^2^ statistics: 9.802; p value: 0.689			
Energy intake (kcal/day)	2,511 ± 605	2,462 ± 734	-516, 615
Protein (g/day)	97 ± 28	92 ± 40	-27, 36
Carbohydrates (g/day)	323 ± 85	324 ± 90	-70, 68
Fat (g/day)	96 ± 31	92 ± 37	-26, 34
Total calcium (mg/day)	1,415 ± 587	1,241 ± 575	-337, 684
Calcium dairy (mg/day)	799 ± 476	679 ± 499	-296, 536
Calcium combination foods (mg/day)	198 ± 144	170 ± 147	-116, 171
Phosphorus (mg/day)	1,678 ± 551	1,544 ± 546	-330, 598
Zinc (mg/day)	14 ± 7	14 ± 6	-6, 6
Vitamin D (mcg/day)	9 ± 6	7 ± 5	-4, 7
Soda beverage (can/day)	1.1 ± 1.0	1.1 ± 1.1	-0.9, 0.9

**Figure 2:**
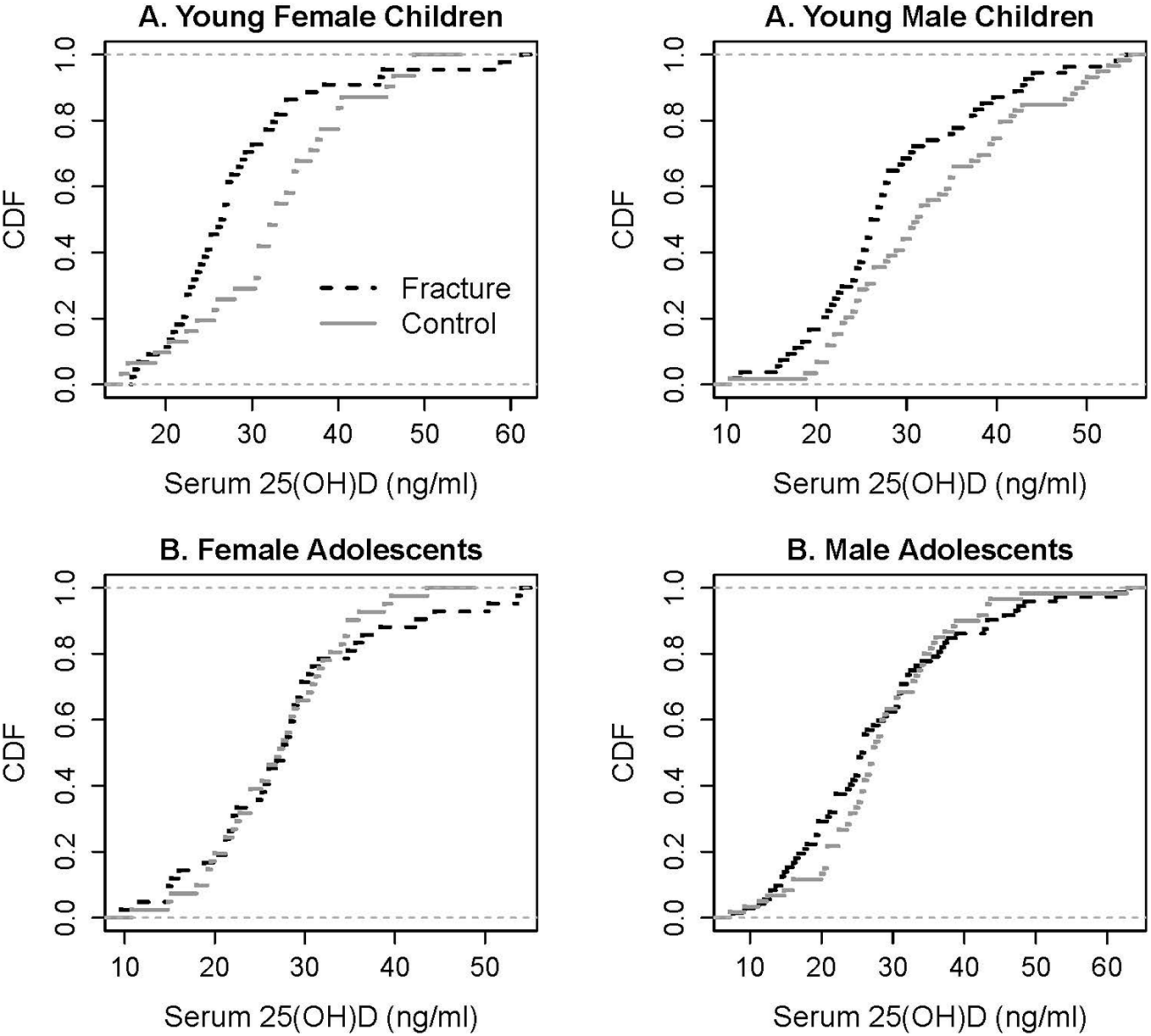
Empirical cumulative distribution functions (cdf) of serum 25(OH)D levels in young children and adolescents with fracture and its controls. Kolmogorov–Smirnov test to quantify the distance between the cdfs of the two samples: significant for young male children (p = 0.0444, test statistic 0.2598) and young female children (p = 0.019, test statistic 0.4369).

Further examination of the vitamin D status among fracture cases and controls by the ecdf/Kolmogorov–Smirnov test (significance level p and test statistic) (**Figure 2**) of serum 25(OH)D levels reveals that there was a higher proportion of male children (p = 0.0444, 0.2598) and female children (p = 0.0019, 0.4369) with fracture with serum 25(OH)D levels below the threshold of 31 ng/ml (39). Around 72% of female and male children with fracture had serum 25(OH)D levels below the threshold, compared with only 42% of female controls and to 51% of male controls. Vitamin D status was the same among male and female adolescents with and without fracture (**Figure 2**), with the average 25(OH)D concentrations of 27.5 ± 11.4, 27.8 ± 10.8, 28.3 ± 9.8, and 26.9 ± 7.2 ng/ml, respectively, all below the threshold. A threshold of 30 ng/ml was recently accepted by the Endocrine Society practice guidelines as a cutoff point for bone health in children and adults (40) and is similar to the one we previously determined (39).

Lower serum levels of 25(OH)D were found in the small cohort of African American children, among either fracture cases (20.1 ± 9.0 ng/ml) and controls (22.6 ± 11.5 ng/ml) as compared with their Caucasian counterparts: fracture group (28.8 ± 10.2 ng/ml) and control group (31.0 ± 8.8 ng/ml) (mean ± SD). However, the observed differences in the mean serum vitamin D levels between the racial groups were not statistically significant (p > 0.05).

## Discussion

The study documented reduced bone mineral density at multiple skeletal regions of interest in both female and male adolescents with bone fragility fracture consistent with the compromised bone integrity of rapid bone modeling. Bone deficiency was more pronounced in women than in men. This was documented at multiple skeletal regions of interest by the DXA, pQCT, and metacarpal radiogrammetry measurements, for both cortical and trabecular bone sites. Adolescent women with fracture had reduced cross-sectional areas of the radius and metacarpals whereas this was not present in adolescent men with fracture. Bone status of young female and male children with fracture was not different compared with their controls. A higher proportion of young children and adolescents with increased body fat were present in the fracture groups. Vitamin D deficiency was more prevalent in young children with fracture as compared with their controls.

The peak incidence of bone fragility fractures in young individuals coincides with the pubertal growth spurt of early adolescence (1, 3, 4); however, most of the studies addressing the issue of bone status during growth did not separate childhood from adolescence (21). Few prospective studies did indicate increased risk of bone fragility during adolescence in children who had reduced bone mineral density during childhood (22, 23, 26). It has been postulated, however, that the dissociation between bone size and mineralization may result in relative weakness in skeletal regions during the adolescent growth spurt (10). Bone modeling of early adolescence is characterized by rapid skeletal expansion along the longitudinal axis with an enlargement of periosteal envelope with compromised bone apposition from within the bone as an organ. Around 37% of the total skeletal mass is accumulated during this developmental phase (32). A mild secondary hyperparathyroidism is present during this stage of skeletal development due to a high demand for calcium, and it is more pronounced in adolescents with lower calcium intake (34). Consequently, serum 1,25-dihydroxyvitamin D concentrations are higher during rapid bone modeling of early adolescence as compared with the rate of bone modeling during childhood and skeletal consolidation of late adolescence (41). When the skeleton reaches maturity by young adulthood, the incidence of fractures due to moderate trauma declines drastically and reaches all time low levels.

In addition, several studies documented smaller bones in children with fracture (18, 20). In this study, adolescent women with fracture had the same stature and skeletal age as the controls; however, they did have a slight reduction in cross-sectional areas of the radius and metacarpals. Narrower forearm bones, in addition to the reduced volumetric bone mineral density, may contribute to bone fragility by itself, due to a lower cross-sectional moment of inertia (18).

This study did confirm a worldwide trend for increased body fatness in children with fracture (15, 17, 18, 20, 42, 43). This in part may explain the secular trends for the rising incidence of the distal forearm fractures among young residents of the Rochester County, Minnesota (8). Obesity, coupled with compromised bone density and smaller bones, can make children more vulnerable to distal forearm fracture following trauma (18). Increased body fat content with higher serum leptin levels leads to an early onset of puberty and may further alter the normal relationship between skeletal growth and mineralization (38).

Lean body mass measurements by representing muscle mass and handgrip strength were not different between the groups. This may only indirectly suggest a similar exposure to physical activity, presumably an independent determinant of fracture during growth. Vigorous physical activity increases fracture risk in children irrespective of bone mass, as shown in a prospective study among healthy children from UK (44).

Vitamin D deficiency was more prominent in young children with fracture as compared with the healthy controls. Vitamin D deficiency can negatively influence muscle power in young individuals (45), and this may indirectly predispose children to falls, as documented in adults (46). However, the clinical significance of this finding is unclear at present as the blood draw was done after the fracture event. A determination of vitamin D status at the time of a fracture and/or an intervention study with vitamin D supplementation is warranted to clarify the significance of this observation. It was previously implicated that latitude may influence childhood fracture (21), and this could be related to the underlying vitamin D status. This particularly applies to the African American children who may be at a higher risk of vitamin D deficiency.

The results of the study have practical implications with regard to the prevention of bone fragility during growth. Preventive measures should specifically target the adolescents, who have the highest rate of bone fragility fractures and whose number has been increasing steadily over the last two decades topping the baby-boom-fueled teen explosion of the 1960s and 1970s. The total number of adolescents aged 12–17 years is currently estimated at 25.1 million with projection to 29.6 million by the year 2030 (www.childtrendsdatabank.org). Secondary to this change, the number of fractures in young individuals has been increasing (8, 9) with the approximate cost of treatment estimated in the USA in 1999 at ~0.5 billion dollars annually (7). Other studies have shown that exogenous factors may influence bone mass during skeletal modeling and, therefore, may play an important role in fracture prevention. Of note, as documented in a 7-year calcium supplementation study among adolescent women, calcium intake up to 1,500 mg/day had a significant impact on bone accretion during bone modeling of pubertal growth spurt with a resultant lower forearm fracture rate due to minimal to moderate trauma (34, 47). In the current study, the total daily calcium intake as well as calcium intake from dairy sources in the study participants was not different between the fracture groups and its controls. However, the dietary interview was conducted *post festum*, which may bias the results, as young patients presumably could have changed their dietary behavior following fracture. Several studies previously documented lower calcium intakes and consumption of dairy products in children with fracture (13, 20, 48, 49). A randomized study of the efficacy of calcium/dairy supplementation with/without vitamin D in fracture prevention during adolescence is needed to confirm the current trends, with the goal to decrease the burden of bone fragility fractures in this segment of the pediatric population (50).

In conclusion, we documented a lower bone mineral density at multiple skeletal regions of interest among adolescents of both sexes but not in younger children. This confirms the presence of deficient bone accretion during rapid skeletal modeling when the incidence of bone fragility fractures is the highest. As the study was conducted in central Ohio with the predominant Caucasian population well represented in the study groups, we cannot extrapolate its findings to the other populations of different ethnic background. Ideally, a long-term, large-scale, follow-up study of bone mass accrual among boys and girls of different ethnicity from an early childhood to young adulthood should be conducted to further evaluate the relationship between bone health and fracture rate.

## Data availability statement

The raw data supporting the conclusions of this article will be made available by the authors, without undue reservation.

## Ethics statement

The studies involving human participants were reviewed and approved by Biomedical Sciences Institutional Review Board, The Ohio State University. Written informed consent to participate in this study was provided by the participants’ legal guardian/next of kin.

## Author contributions

All authors contributed to the article and approved the submitted version.
